# CAT: Centerness-Aware Anchor-Free Tracker

**DOI:** 10.3390/s22010354

**Published:** 2022-01-04

**Authors:** Haoyi Ma, Scott T. Acton, Zongli Lin

**Affiliations:** Charles L. Brown Department of Electrical and Computer Engineering, University of Virginia, Charlottesville, VA 22904-4743, USA; hm5au@virginia.edu (H.M.); zl5y@virginia.edu (Z.L.)

**Keywords:** visual object tracking, anchor-free, centerness, convolutional neural network

## Abstract

Accurate and robust scale estimation in visual object tracking is a challenging task. To obtain a scale estimation of the target object, most methods rely either on a multi-scale searching scheme or on refining a set of predefined anchor boxes. These methods require heuristically selected parameters, such as scale factors of the multi-scale searching scheme, or sizes and aspect ratios of the predefined candidate anchor boxes. On the contrary, a centerness-aware anchor-free tracker (CAT) is designed in this work. First, the location and scale of the target object are predicted in an anchor-free fashion by decomposing tracking into parallel classification and regression problems. The proposed anchor-free design obviates the need for hyperparameters related to the anchor boxes, making CAT more generic and flexible. Second, the proposed centerness-aware classification branch can identify the foreground from the background while predicting the normalized distance from the location within the foreground to the target center, i.e., the centerness. The proposed centerness-aware classification branch improves the tracking accuracy and robustness significantly by suppressing low-quality state estimates. The experiments show that our centerness-aware anchor-free tracker, with its appealing features, achieves salient performance in a wide variety of tracking scenarios.

## 1. Introduction

Visual object tracking (VOT) is a crucial vision task with numerous applications, such as medical imaging, defense, video security camera systems, autonomous vehicles, and robotics [1,2,3,4]. In a video sequence, given the target state in the first frame, the goal of visual object tracking is to estimate the target state in the subsequent frames. Although remarkable progress has been made, VOT is challenging because the target object may experience extreme appearance variations resulting from interfering challenging factors such as motion blur and deformation.

Recently, visual trackers based on a Siamese network have received considerable attention due to the balanced accuracy and speed. In the pioneering work, a fully convolutional Siamese network (SiamFC) [5] is achieved via training a Siamese network to perform template matching and compute a similarity score between the exemplar image of the target object and the search image. In order to adjust to scale variation, a multi-scale searching scheme is employed as in [6,7] to perform template matching on multiple scales to identify the best scale. However, the multi-scale searching scheme is computationally expensive when facing large scale variations due to the multi-scale feature extraction step. To address this issue, the Siamese region proposal network (SiamRPN) [8] employs a regional proposal network (RPN) [9] that includes a classification branch to identify the foreground and a regression branch to refine the predefined candidate anchor boxes. This representative anchor-based method avoids the time-consuming multi-scale feature extraction step in the multi-scale searching scheme while achieving promising accuracy. However, as shown in [9,10], to achieve satisfactory performance, the hyperparameters related to the anchor boxes (e.g., numbers, sizes, and aspect ratios) entail tedious and heuristic tuning.

To obviate the need for an anchor box and the associated parameters, the fully convolutional Siamese network for classification and regression (SiamCAR) [11] decomposes the tracking into three sub-problems, a binary classification problem, a centerness estimation problem, and a regression problem. However, to obtain the products of the centerness scores and the binary classification scores, instead of training a branch to directly output the products, two separate branches are trained for centerness estimation and binary classification, respectively, and aggregated in the inference stage, which may yield an inconsistency risk (to be detailed in Section 3.5) between the training and the inference stages, and therefore may result in less ideal tracking performance.

In this paper, we design a centerness-aware anchor-free tracker (CAT) by decomposing the tracking into parallel classification and regression problems. With the above decomposition, the tracking problem is addressed in a per-pixel fashion. The regression branch regresses each spatial location a relative bounding box by predicting the distance from the corresponding spatial location to each side of the axis-aligned ground truth bounding box. Based on the observation that many low quality predictions generated correspond to the locations that are far away from the center of the target object, the classification branch is designed to learn to output a 0 for the background and a value ranging from 0 to 1 to indicate the normalized distance between the spatial location within the foreground and the target center, thus suppressing low quality state estimations. The main contributions of this article can be summarized as follows. First, within the anchor-free tracking scheme, we propose a centerness-aware classification branch that acts as a joint representation of the binary classification and the centerness estimation branches, and the resulting system is named CAT. Second, the proposed centerness-aware classification branch mitigates the inconsistency risk and improves the tracking performance with reduced computation cost and fewer associated parameters. Third, proof of validation and the efficacy of CAT is achieved through extensive experiments that are performed on publicly available benchmark datasets.

The organization of this article is described as follows. In Section 2, Siamese-network-based trackers and anchor-free mechanisms that are most relevant to our approach are reviewed. In Section 3.1, Section 3.2, Section 3.3, we describe the main components of the proposed network including the feature extraction, the feature combination, and the state estimation components. The details about the loss function are described in Section 3.4. The relationship between prior anchor-free methods and our proposed tracker are detailed in Section 3.5. In Section 4.1, the implementation details of the training and inference stages are described. The benchmark datasets and evaluation metrics are detailed in Section 4.2. In Section 4.3, the qualitative and quantitative comparison results are presented. In Section 4.4, an ablation test is performed to reveal the contribution of each component. Section 5 concludes the article.

## 2. Related Work

In this section, we review visual trackers based on the Siamese network and anchor-free mechanism that are closely related to our proposed tracker.

### 2.1. Siamese Network-Based Trackers

In recent years, the Siamese network has been extensively used in the tracking community because of its balanced accuracy and efficiency. In the pioneering work of SiamFC [5], the Siamese network is employed for feature extraction, and a cross-correlation layer is used to calculate a similarity score between the target and candidate image patches. Furthermore, a transformation learning model is proposed in the dynamic Siamese network (DSiam) [12] to learn the appearance change of the target object more effectively and to suppress the background. In the residual attentional Siamese network (RASNet) [13], various attention mechanisms are incorporated for better adaption to the current target state. The Siamese network with semantic and appearance branches (SA-Siam) [14] achieves improved performance through training the two branches separately so that the heterogeneity of different types of features can be retained. Although promising results have been achieved, these methods resort to the multi-scale searching scheme, and cannot accommodate large scale variations and aspect ratio variations.

To tackle the large scale variation and aspect ratio variations through refining the predefined candidate anchor boxes, SiamRPN [8] combines SiamFC [5] with an RPN [9] and obviates the need for the time-consuming step of extracting feature maps at multiple scales. Furthermore, the series-parallel matching real-time tracker (SPM-tracker) [15] improves the robustness and discrimination power of SiamRPN via a series-parallel matching scheme. Through increasing the hard negative training data in the training stage, the distractor-aware Siamese regional proposal network (DaSiamRPN) [16] improves the robustness of the model. To further improve the accuracy, an effective sampling strategy is designed in the Siamese tracking with a deep network (SiamRPN++) [17] to remove the center bias in the training stage, enabling the extraction of features using deeper neural networks. However, as shown in [9,10], the hyperparameters related to the anchor boxes require heuristic tuning to achieve good performance. In contrast, CAT addresses VOT in an anchor-free manner and obviates the tuning related to the hyperparameters of the anchor boxes and hence is more flexible.

### 2.2. Anchor-Free Mechanism

Recently, anchor-free methods have been widely employed in a family of object detectors due to their simple architectures and promising performance. In contrast to the anchor-based methods that refine the predefined candidate anchor boxes, the anchor-free methods estimate the target state in a more direct way. Generally, the anchor-free detectors can be grouped into keypoint-based object detectors and dense object detectors.

In Dense RepPoints [18], the object is represented as a large set of keypoints to describe the object at multiple levels. On the other hand, in CornerNet [19], the target state is described as paired keypoints and detected using a single convolution neural network. Similar to CornerNet, the target state is represented as four extreme points (top-most, left-most, bottom-most, right-most), together with one center point in ExtremeNet [20], and detected using a standard keypoint estimation network. Based on CornerNet, CenterNet [21] proposes to exploit the global information of the object to improve the performance. In [22], the object is modeled as a single point (the center), and all other object proprieties (e.g., size and orientation) are then regressed directly from image features corresponding to the center location. In the feature-selective anchor-free object detector (FSAF) [23], a feature-selective module is proposed to address the heuristic-guided feature selection and overlap-based anchor sampling problems that exist in the conventional anchor-based detectors.

In contrast to the keypoint-based methods, FoveaBox in [24] learns the object and the bounding box coordinates without any references (e.g., anchors or keypoints). Furthermore, the fully convolutional one-stage object detector (FCOS) treats the object detection as a binary foreground-background classification for each spatial location, together with the regression of the relative bounding box for the corresponding location. In addition, the centerness branch is proposed to suppress low quality predictions corresponding to the locations that are far from the target center. In VarifocalNet [25], a new loss function and a new star-shaped bounding box feature representation are proposed to improve the ranking and refinement of the bounding boxes. In tracking, to remedy the issue, in an anchor-based method, of it being difficult to refine the anchors whose overlap with the target object is small, Ocean is proposed in [26], which focuses on how to enable the rectification of imprecise bounding box predictions and learns an object-aware feature to further enhance the matching accuracy. Similar to FCOS, SiamCAR [11] is proposed to address the tracking problem by decomposing the tracking task into one classification problem, one centerness estimation problem, and one regression problem. Our work is inspired by both FCOS and SiamCAR but is different from these two works. The key differences will be discussed in detail in Section 3.5.

## 3. Proposed Algorithm

**Figure 1:**
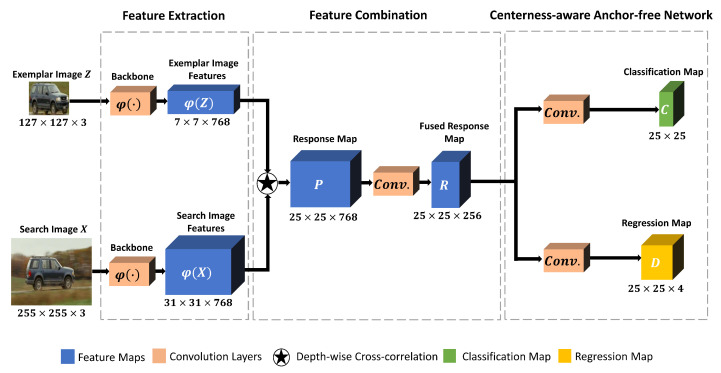
Flowchart of the proposed CAT tracker. The backbone Siamese network takes exemplar image *Z* and search image *X* as input and outputs corresponding feature maps denoted as φ(Z) and φ(X). In order to embed the features from two branches, a depth-wise cross-correlation operation is employed to obtain the multi-channel response map denoted as *P*. Then, to reduce the computation, a convolution layer with a kernel size of 1×1 is employed to fuse the response map. The fused response map with reduced dimension is denoted as *R* and is adopted as the input to the centerness-aware anchor-free network. Regarding every spatial location on the regression map *D*, the regression branch learns to estimate the distance from the corresponding location to each side of the ground truth bounding box. For the classification map *C*, with the observation that many low-quality predictions are produced corresponding to the locations far from the target center, the centerness-aware classification branch learns to output a 0 for the background and a value ranging from 0 to 1 to indicate the normalized distance between the spatial location within the foreground and the target center to suppress predictions with low quality.

In this section, the details of our proposed tracker CAT are described. As shown in Figure 1, CAT is built upon three main components: a Siamese network as a feature extractor, a convolution layer with a kernel size of 1×1 after a depth-wise cross-correlation operation for feature combination, and a centerness-aware anchor-free network for state estimation.

### 3.1. Feature Extraction

The Siamese network takes an exemplar image and a search image as input. The image patch with the center point as the target center is cropped in the initial frame, and is then adopted as the exemplar image. The search images are cropped in the following frames to denote the search region. The Siamese network includes two branches that share the same weights to embed both patches into the same feature space. The Siamese network takes the exemplar image *Z* and the search image *X* as input and outputs φ(Z) and φ(X) as the corresponding feature maps.

As in [11,17], a modified ResNet-50 [27] is used for feature extraction. Specifically, the strides of the last two residual blocks are reduced to eight, and thus the last three residual blocks output feature maps with the same spatial resolution. Moreover, an extra 1×1 convolution layer is appended to each of the block outputs to reduce the channel number to 256. We aggregate hierarchical features as in [11] to achieve higher robustness and accuracy. Formally, the features generated from the last three residual blocks are respectively denoted as $f_{3}\left(\cdot \right)$, $f_{4}\left(\cdot \right)$, and $f_{5}\left(\cdot \right)$ and combined as

(1) $$\varphi \left(\cdot \right)=f_{3}\left(\cdot \right)+\;+\;f_{4}\left(\cdot \right)+\;+\;f_{5}\left(\cdot \right),$$

where ++ is the concatenation operation; $f_{3}\left(\cdot \right)$, $f_{4}\left(\cdot \right)$, and $f_{5}\left(\cdot \right)$ each include 256 channels; and φ(·) consists of 768 channels.

### 3.2. Feature Combination

A depth-wise cross-correlation layer is employed here as in [11,17] to embed the features from the two branches and generate the multi-channel response map *P* as

(2) $$P_{k}=\varphi_{k}\left(Z\right)★\varphi_{k}\left(X\right),$$

where ★ represents the cross-correlation operation, and $\varphi_{k}\left(Z\right)$, $\varphi_{k}\left(X\right)$, and $P_{k}$ denote the *k*-th channel of φ(Z), φ(X), and *P*, respectively. Furthermore, a 1×1 convolution layer is employed to fuse the response map and reduce the dimension from 768 channels to 256 channels. Through the dimension reduction operation, the number of parameters can be reduced to speed up the following computation. The response map with the reduced dimension is denoted as *R* and employed as the input to the centerness-aware anchor-free network.

### 3.3. Centerness-Aware Anchor-Free Network

The centerness-aware anchor-free network includes two branches: a regression branch and a classification branch that is centerness aware. As in Figure 1, regarding the input *R*, the centerness-aware classification branch produces the classification map $C_{w\times h}$, and the regression branch produces the regression map $D_{w\times h\times 4}$, where *w* and *h* denote the width and height of the classification and regression maps. Each location (m,n) on $C_{w\times h}$ or $D_{w\times h\times 4}$ corresponds to one spatial location on the search image *X* as $\left(p_{m},p_{n}\right)=\left(\left\lfloor \frac{s}{2}\right\rfloor +m\times s,\left\lfloor \frac{s}{2}\right\rfloor +n\times s\right)$, where *s* denotes the network stride and ⌊⌋ represents the floor operation. Compared to the anchor-based methods that take every location as the center point of a set of predefined anchor boxes and predict the offset values regarding the anchor boxes, we predict the target bounding box for that spatial location directly.

The regression branch learns to output the distance from the spatial location to each side of the ground truth bounding box. Let the width, height, center point, upper-left corner, and lower-right corner be $g_{w}$, $g_{h}$, $\left(g_{x_{c}},g_{y_{c}}\right)$, $\left(g_{x_{ul}},g_{y_{ul}}\right)$, and $\left(g_{x_{lr}},g_{y_{lr}}\right)$, respectively. The regression targets $D_{m,n}^{t}=\left(l_{m,n},t_{m,n},r_{m,n},b_{m,n}\right)$ for location (m,n) are computed as

(3) $$\begin{array}{c} l_{m,n}=p_{m}-g_{x_{ul}},t_{m,n}=p_{n}-g_{y_{ul}},\\ r_{m,n}=g_{x_{lr}}-p_{m},b_{m,n}=g_{y_{lr}}-p_{n}, \end{array}$$

where $l_{m,n}$, $t_{m,n}$, $r_{m,n}$, and $b_{m,n}$ denote the distance from $\left(p_{m},p_{n}\right)$ to the left side, top side, right side, and bottom side of the ground truth bounding box, respectively.

Different from the centerness branch in SiamCAR [11] that only learns to output centerness value for the foreground spatial locations and generate uncontrollable values for background spatial locations, our proposed centerness-aware classification branch acts as the joint representation of the binary classification and the centerness branches that learns to differentiate the foreground from the background and to output the centerness value for the foreground spatial locations to denote the normalized distance between the spatial location within the foreground and the target center. Specifically, the target value $C_{m,n}^{t}$ for location (m,n) is set to be 0 for the background and ranges from 0 to 1 for the foreground to denote the normalized distance from location $\left(p_{m},p_{n}\right)$ to the target center. Formally, $C_{m,n}^{t}$ is defined as

(4) $$C_{m,n}^{t}={𝟙}_{c}\left(D_{m,n}^{t}\right)\times \sqrt{\frac{\min(l_{m,n},r_{m,n})}{\max(l_{m,n},r_{m,n})}\times \frac{\min(t_{m,n},b_{m,n})}{\max(t_{m,n},b_{m,n})}},$$

where ${𝟙}_{c}$ is the indicator function defined as

(5) $${𝟙}_{c}\left(D_{m,n}^{t}\right)=\left\{\begin{array}{cc} 1, & if\min(D_{m,n}^{t})>0,\\ 0, & \mathrm{otherwise}. \end{array}\right.$$

The regression branch is composed of four convolutional layers with a kernel size of 3×3 and a channel number as 256 and one convolutional layer with the same kernel size and a channel number of 4. Then, an exponential operation is used to map the real number output into (0,+∞) because the target values of the regression branch output are positive real numbers. The centerness-aware classification branch has the same structure with the only difference being that the channel number of the last convolutional layer is 1 instead of 4. Then, the output will be mapped into (0,1) through a sigmoid function.

### 3.4. Loss Function

We adopt the intersection over union (IoU) loss [28] for the regression branch. Here, we only treat the locations within an elliptical area centered within the ground truth bounding box as positive samples as in [29]. More concretely, the regression loss $L_{\mathrm{reg}}$ is defined as

(6) $$L_{\mathrm{reg}}=\frac{-1}{\sum_{m,n}{𝟙}_{d}\left(D_{m,n}^{t}\right)}\sum_{m,n}{𝟙}_{d}\left(D_{m,n}^{t}\right)\times \ln\left(IoU(GT,B_{m,n})\right),$$

where GT and $B_{m,n}$ denote the ground truth bounding box and the predicted bounding box corresponding to location (m,n), and ${𝟙}_{d}\left(D_{m,n}^{t}\right)$ is the indicator function defined as

(7) $${𝟙}_{d}\left(D_{m,n}^{t}\right)=\left\{\begin{array}{cc} 1, & if{\left(\frac{p_{m}-g_{x_{c}}}{\alpha \times g_{w}}\right)}^{2}+{\left(\frac{p_{n}-g_{y_{c}}}{\alpha \times g_{h}}\right)}^{2}<1,\\ 0, & otherwise, \end{array}\right.$$

with α controlling the size of the area corresponding to the positive samples in the training of the regression branch.

Acting as a joint representation of the binary classification and centerness estimation branches, the proposed centerness-aware classification branch does pose additional challenges to the training process. The training of the proposed centerness-aware classification branch needs dense supervisions across the whole search image and hence may result in a data imbalance problem. Moreover, compared to the binary classification branch in SiamCAR that only contains discrete labels, the continuous labels ranging from 0 to 1 make the training more challenging. As shown in Section 4.4, the standard cross-entropy loss cannot successfully train the proposed centerness-aware classification branch due to challenges in the training process. Therefore, to ensure the successful training, we combine the cross-entropy loss [30] with the focal loss [10] as

(8) $$\begin{array}{cc} L_{c} & =\frac{-1}{\sum_{m,n}{𝟙}_{d}\left(D_{m,n}^{t}\right)}\sum_{m,n}{\left|C_{m,n}-C_{m,n}^{t}\right|}^{\beta }\\ \; & \times \left(C_{m,n}^{t}\times \ln\left(C_{m,n}\right)+\left(1-C_{m,n}^{t}\right)\times \ln\left(1-C_{m,n}\right)\right), \end{array}$$

where $-\left(C_{m,n}^{t}\times \ln\left(C_{m,n}\right)+\left(1-C_{m,n}^{t}\right)\times \ln\left(1-C_{m,n}\right)\right)$ acts as the cross-entropy part. Similar to [10], the modulating factor ${\left|C_{m,n}-C_{m,n}^{t}\right|}^{\beta }$ reflects the difference between the estimation $C_{m,n}$ and the continuous label $C_{m,n}^{t}$ and makes the training process focus more on the hard samples via reducing the loss contribution of the easy samples, and β balances the importance of easy and hard samples. Hence, the training of the entire network is to minimize the multi-task loss defined as

(9) $$L_{\mathrm{total}}=\lambda_{1}L_{c}+\lambda_{2}L_{\mathrm{reg}},$$

where $\lambda_{1}$ and $\lambda_{2}$ are the tradeoff parameters that balance the importance of $L_{c}$ and $L_{\mathrm{reg}}$.

### 3.5. Relationship to Prior Anchor-Free Work

CAT shares some motivating factors with the recent detection method FCOS [29] and the recent tracking method SiamCAR [11]. Here, we discuss the differences with respect to these two related works.

In FCOS, the task is to detect the category of the objects, and the categories are predefined. In contrast, in VOT, it is necessary to determine the object at the instance level. Hence, in CAT, a template branch is adopted to encode the target object appearance information. Both FCOS and SiamCAR use a binary classification branch to identify foreground from background and a branch to predict the centerness value to indicate the normalized distance from the corresponding spatial location to the target center. The low-quality predictions that are far from the target center are suppressed via multiplying the outputs of the centerness branch with those of the binary classification branch. However, it should be noted that, in both the FCOS and SiamCAR methods, the centerness branch is only trained with respect to the centerness value corresponding to spatial locations within the groundtruth bounding box, as the centerness value is not defined for background spatial locations. Hence, in the centerness branch, the output for the background spatial location is unconstrained. This will not create any trouble in the most ideal scenario, where the binary classification branch is perfectly trained and outputs exact 0 and 1 for background and foreground spatial locations, respectively. However, in the inference stage, the binary classification branch will not output exact 0 and 1 and may result in an inconsistency risk. For example, when the background classification score is not 0 and the foreground classification score is not high enough, it is possible that the state estimation corresponding to background spatial locations is chosen as the tracking result due to the high unconstrained centerness score corresponding to the background spatial locations. In addition, since the regression branch is only trained with respect to the foreground spatial locations, using the output of the regression branch that corresponds to the background spatial locations cannot provide an accurate state estimation of the target object and may result in tracking failures. Based on these observations, we propose the centerness-aware classification branch to directly learn the products of binary classification branch outputs and the centerness branch outputs in SiamCAR to mitigate the inconsistency risk between the training and inference stages. Different from the centerness branch in SiamCAR [11] and FCOS [29], our proposed centerness-aware classification branch will act as the joint representation of the binary classification branch and the centerness estimation branch to output 0 for the background and the centerness value for the foreground, hence mitigating the inconsistency risk and improving the tracking performance with reduced computation cost and fewer associated parameters.

## 4. Experiments

Extensive experiments have been performed on benchmark datasets to verify the efficacy of our proposed tracker. The implementation details about training and inference are presented in Section 4.1.1 and Section 4.1.2, respectively. Section 4.2 describes the employed benchmark datasets and the evaluation protocols. Qualitative and quantitative comparison results between our tracker and other representative trackers are presented in Section 4.3. In Section 4.4, we perform an ablation test to show the contribution of each component.

### 4.1. Implementation Details

#### 4.1.1. Training

As in [5], the exemplar image size is set as 127×127 pixels, and the search image size is set as 255×255 pixels. The training datasets include COCO [31], YouTube-BB [32], ImageNet VID and DET [33], and GOT-10k [34]. The weights of the backbone Siamese network are set as the pretrained weights on ImageNet as in [17]. We train the entire network via stochastic gradient descent, in which the minibatch size is set as 64 and the number of epochs is set to 20 as in [11]. The learning rate will be increased from 0.001 to 0.005 using a warm-up strategy in the first five epochs, and will be decayed from 0.005 to 0.0005 in the remaining 15 epochs as in [11]. The weights of the backbone Siamese network is fixed in the first half training stage and fine-tuned with one-tenth of the present learning rate for the remaining epochs as in [11,17]. The values of the momentum and the weight decay are set as 0.9 and $10^{-3}$, respectively. The parameters α, β, $\lambda_{1}$, and $\lambda_{2}$ are tuned manually, using the best-performing values of 0.6, 2, 2, and 6, respectively. Our approach is implemented in Python using PyTorch on a PC with Intel Core i9-9820X CPU 3.30 GHz, 64GB RAM, two NVIDIA GeForce RTX 2080 Ti GPUs, and one NVIDIA GeForce GTX TITAN V GPU.

#### 4.1.2. Inference

During inference, we adopt the offline tracking strategy as in [11,17]. The features of the exemplar image are computed only in the first frame and used to match the subsequent search images. The search image is cropped centered on the previous estimated target center. The predicted bounding box $B_{m,n}$ for location (m,n) is computed as

(10) $$\begin{array}{c} b_{x_{ul}}=p_{m}-D_{m,n,0},b_{y_{ul}}=p_{n}-D_{m,n,1},\\ b_{x_{lr}}=p_{m}+D_{m,n,2},b_{y_{lr}}=p_{n}+D_{m,n,3}, \end{array}$$

where $\left(b_{x_{ul}},b_{y_{ul}}\right)$ and $\left(b_{x_{lr}},b_{y_{lr}}\right)$ represent the upper-left corner and the lower-right corner coordinates of the predicted bounding box $B_{m,n}$. As in [11,17], a scale change penalty and Hanning window are used to suppress extreme scale variation and fast motion and to obtain the final classification map. In the final classification map, the maximum value suggests the location of the target object. To maintain a smooth change of the shape of the bounding box prediction, the estimated size is updated through linear interpolation with the estimated size from the last frame.

### 4.2. Datasets and Evaluation Metrics

All the experiments are performed on the benchmark datasets visual object tracking challenge 2019 (VOT-2019) [35] and unmanned aerial vehicle dataset (UAV123) [36]. The UAV123 dataset contains 123 challenging aerial videos with more than 110K frames captured from low-altitude UAVs. For UAV123, precision and success rate are used as the evaluation metrics to quantitatively evaluate the tracker performance. The precision score is defined as the fraction of total frames where the center location error (CLE) between the predicted target center and the ground truth target center is within the specified threshold distance. As in the evaluation toolkit [36], the precision score at the threshold of 20 pixels is used as the representative precision score (Prec.). The success rate is defined as the fraction of total frames where the intersection over union (IOU) between the predicted and ground truth bounding boxes is above a predefined threshold. The area-under-curve (AUC) score is reported to summarize the success plot generated by computing fractions of successful frames at different defined thresholds of IOU.

VOT-2019 contains 60 challenging videos. Here, the tracker is reinitialized when the tracker drifts off target. Three metrics are employed: accuracy (A), robustness (R), and expected average overlap (EAO). The accuracy denotes the mean IOU between the predicted bounding box and the ground truth bounding box throughout successful tracking intervals. The robustness denotes the number of times that the target object is lost per video sequence. The EAO denotes the expected no-reset overlap (IOU) between the predicted and the ground truth bounding boxes, and is the primary measure that is used to summarize the performance of the tracker in VOT-2019 since it considers both the accuracy and the robustness.

### 4.3. State-of-the-Art Comparison

To validate the performance of the proposed tracker, CAT is compared to five representative state-of-the-art trackers. Based on the methods employed to address the scale estimation, the selected trackers can be broadly categorized as follows:

- SiamFC [5] employs the multi-scale searching scheme to perform template matching on multiple scales to identify the best scale.
- SiamRPN [8] and SiamRPN++ [17] employ an anchor-based method to obtain the state estimation.
- SiamCAR [11] addresses the tracking problem in an anchor-free approach.
- SiamMask [37] represents the target object as a binary segmentation mask instead of axis-aligned bounding boxes.

**Figure 2:**
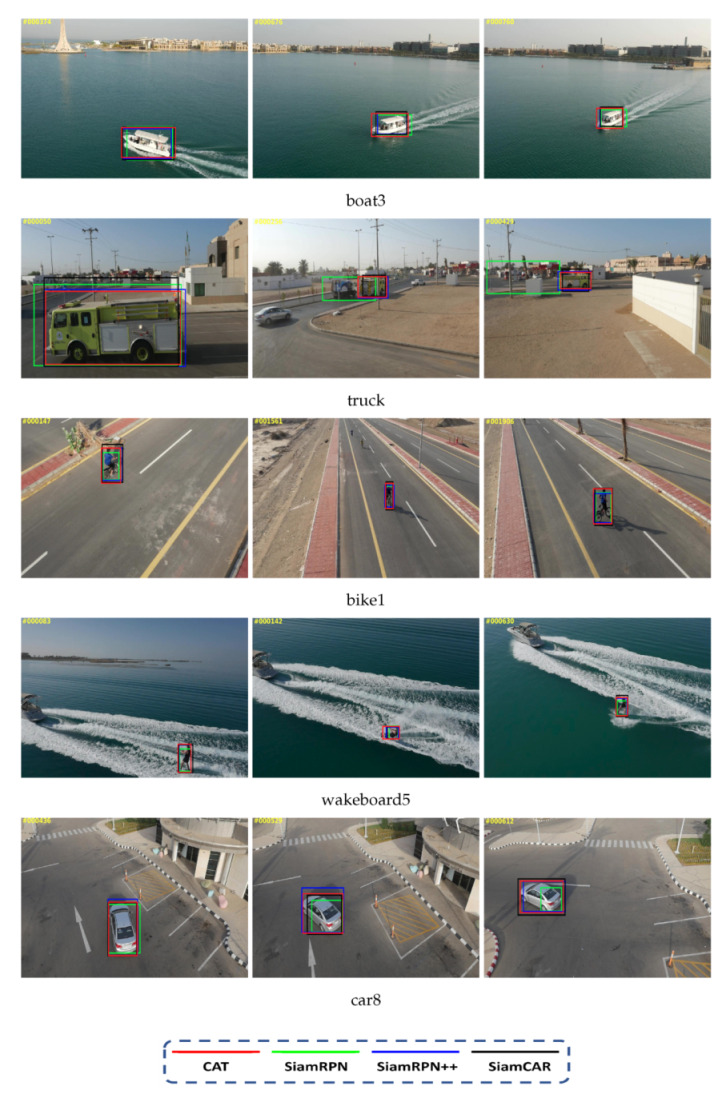
Qualitative comparisons between the proposed tracker CAT and representative trackers SiamRPN [8], SiamRPN++ [17], and SiamCAR [11] on boat3 (first row), truck1 (second row), bike1 (third row), wakeboard5 (fourth row), and car8 (bottom row) sequences that involve large scale variations and aspect ratio variations. Compared to other trackers, even facing challenging scenarios including occlusion, large scale variations, and aspect ratio variations, CAT provides accurate state estimations that significantly improve the robustness and accuracy in tracking.

All trackers are evaluated on UAV123 and VOT-2019. Figure 2 and Table 1 and Table 2 summarize the corresponding results. Figure 2 shows qualitative comparison results between our proposed tracker CAT and some representative state-of-the-art trackers. As shown in Figure 2, even when facing partial occlusion, large scale variations, and aspect ratio variations, our tracker provides accurate state estimation, which validates the robustness and accuracy of our approach.

As in Table 1, our proposed tracker CAT obtains the best performance on UAV123 in terms of both AUC score and Prec. score. Compared to SiamCAR that achieves the second best results, CAT outperforms SiamCAR in terms of the AUC score and Prec. score by 2.1% and 2.5%, respectively.

According to Table 2, CAT obtains the best performance in terms of both EAO score and robustness score on VOT-2019. Compared to SiamRPN++, which obtains the best accuracy score, CAT underperforms in terms of the accuracy score by 1.6% but outperforms in terms of the EAO score and the robustness score by 3.2% and 6.6%, respectively. SiamRPN++ obtains the best accuracy score at the cost of a low robustness score due to the tracking reinitialization upon tracking failure in VOT-2019. Compared to SiamCAR, which obtains the second best performance in terms of the EAO score, the accuracy score, and the robustness score, CAT improves the performance by 2.9% and 3.5% on EAO score and robustness score, respectively, while underperforming by only 1.0% on accuracy. These results are indicative of the efficacy and robustness of the proposed anchor-free centerness-aware tracking scheme.

As shown in Table 1 and Table 2, in comparison with SiamCAR, our proposed centerness-aware classification branch mitigates the inconsistency risk and provides promising performance improvement. To further validate the efficiency of our proposed method, we performed the computation and speed analysis, and the average speed (frames per second) on UAV123 is reported in Table 3. As shown in Table 3, compared to SiamCAR [11], our proposed tracker CAT operates with reduced computation cost and fewer associated parameters.

### 4.4. Component-Wise Analysis of the Proposed Method

In order to reveal the contributions of each part of CAT, we design three variants of CAT and perform a component-wise analysis on UAV123 and VOT-2019. In these variants, the regression branches for state estimation are kept the same and trained using the intersection over union loss, and other branches are trained with standard cross-entropy loss if not specifically mentioned. The three variants of our proposed tracker are described as follows, and Table 4 and Table 5 summarize the results.

- CAT_wo_cen: The CAT tracker without the centerness-aware classification branch is denoted as CAT_wo_cen, where a classification branch trained for binary foreground-background identification is employed instead of our proposed centerness-aware classification branch.
- CAT_w_cen_div: The CAT tracker with a separate branch trained for centerness value estimation is denoted as CAT_w_cen_div, in which a single-layer branch paralleling the binary classification branch is trained to estimate the centerness value.
- CAT_wo_mod: The CAT tracker with the proposed centerness-aware classification branch trained by the standard cross entropy loss without the modulating factor.

As presented in Table 4 and Table 5, the centerness branch yields a significant gain, i.e., 13.8 points on the AUC score, 15.9 points on Prec. score, 6.7 points on the EAO score, 11.5 points on the accuracy score, and 3.6 points on the robustness score (CAT_w_cen_div vs. CAT_wo_cen), which verifies the effectiveness of the centerness. By suppressing the low quality predictions, the tracker avoids low quality predictions that may result in tracking failures due to the error accumulation. As shown in the comparison between CAT and CAT_wo_mod, facing the challenges in the training process as described in Section 3.4, the loss function that combines the standard cross-entropy loss and the modulating factor ensures the successful training of our tracker with the proposed centerness-aware classification branch and yields a significant gain. As presented in the comparison between CAT and CAT_w_cen_div, the proposed centerness-aware classification branch brings about a promising performance improvement of 1.7 points on the AUC score, 3.3 points on the Prec. score, 2.6 points on the EAO score, and 3.0 points on the robustness score (CAT vs. CAT_w_cen_div). This result demonstrates that the proposed centerness-aware classification branch mitigates the inconsistency between the training and the inference stages by acting as a joint representation of the binary classification branch and the centerness branch. These comparisons verify the efficacy of different components of CAT.

## 5. Conclusions

In this work, we design an effective centerness-aware anchor-free tracking framework that avoids the heuristic design associated with anchor boxes and a fixed set of scale factors. Based upon the observation that the location near the center of the target can provide high quality predictions, we propose a centerness-aware classification branch to yield a joint representation of the binary classification and the centerness estimation to select high quality predictions. The centerness-aware design mitigates the risk of inconsistency in the training and inference stages. Comprehensive experiments validate the efficacy and the robustness of the resulting tracker, CAT.

To demonstrate the generalizability of our proposed algorithm, our future work will include tracking moving cells in time-lapse video sequences to help understand the mechanisms of cell motility and their regulation, and tracking surgical instruments that are crucial to computer-assisted interventions for minimally invasive surgery. With respect to cell tracking in vitro and in vivo, we anticipate that CAT will afford a generalized, robust cell tracker that can adapt to a range of scales. In surgical applications, CAT can be employed without modification to multiple tools over a range of viewpoints and scales.

## Figures and Tables

**Table 1:** Comparisons of CAT and five representative state-of-the-art methods on UAV123. The best and the second best values are in bold and underlined, respectively. CAT obtains the best performance in terms of the area-under-curve (AUC) and the precision measures. ↑ means that a higher score is better, and ↓ denotes that a lower value is better.

	SiamFC	SiamRPN	SiamMask	SiamRPN++	SiamCAR	CAT
AUC ↑	0.485	0.557	0.603	0.610	0.614	**0.635**
Prec. ↑	0.693	0.768	0.795	0.803	0.813	**0.838**

**Table 2:** Comparisons of CAT with five representative state-of-the-art methods on VOT-2019. The best and the second best values are in bold and underlined, respectively. The EAO score measures the expected no-reset IOU between the estimated bounding box and the ground truth bounding box. The accuracy (A) denotes the mean IOU between the predicted bounding box and the ground truth bounding box in successful tracking intervals. The robustness (R) denotes the number of times that the target is lost per video sequence. ↑ means that a higher score is better, and ↓ denotes that a lower value is better.

	SiamFC	SiamRPN	SiamMask	SiamRPN++	SiamCAR	CAT
EAO ↑	0.189	0.272	0.287	0.285	0.288	**0.317**
A ↑	0.510	0.582	0.592	**0.599**	0.593	0.583
R ↓	0.958	0.527	0.461	0.482	0.451	**0.416**

**Table 3:** Comparisons between CAT and SiamCAR with respect to the number of parameters and speed. ↑ means that a higher score is better, and ↓ denotes that a lower value is better.

	SiamCAR	CAT
Number of Parameters ↓	51,384,903	51,380,293
Speed (Frames Per Second) ↑	54.62	57.83

**Table 4:** Comparisons between CAT and two variants of CAT on UAV123. The best and the second best scores are in boldface and underlined, respectively. ↑ means a higher score is better, and ↓ denotes a lower value is better.

	CAT_wo_cen	CAT_w_cen_div	CAT_wo_mod	CAT
AUC ↑	0.480	0.618	0.595	**0.635**
Prec. ↑	0.646	0.805	0.788	**0.838**

**Table 5:** Comparisons between CAT and two variants of CAT on VOT-2019. The best and the second best scores are in boldface and underlined, respectively. ↑ means that a higher score is better, and ↓ denotes that a lower value is better.

	CAT_wo_cen	CAT_w_cen_div	CAT_wo_mod	CAT
EAO ↑	0.224	0.291	0.266	**0.317**
A ↑	0.475	**0.590**	0.580	0.583
R ↓	0.482	0.446	0.547	**0.416**

## Data Availability

The publicly available benchmark datasets we use can be accessed at https://cemse.kaust.edu.sa/ivul/uav123 and https://www.votchallenge.net/vot2019/ (accessed on 1 February 2021).
